# Value of Wassermann Test

**Published:** 1914-08

**Authors:** 


**Value of Wassermann Test.** Nicolas and Gate, Coc. Med. des Hop. de Lyon, April 7, 1914, claim the following results in 40 plainly syphilitic cases: 60% positive in the period of the chancre; 90% in the secondary period; 80% in the tertiary stage; 36% in parasyphilis. 63 non-syphilitics reacted positively in 30% of the cases.

